# Geographic variations in involuntary care and associations with the supply of health and social care: results from a nationwide study

**DOI:** 10.1186/s12913-018-3064-3

**Published:** 2018-04-06

**Authors:** Coralie Gandré, Jeanne Gervaix, Julien Thillard, Jean-Marc Macé, Jean-Luc Roelandt, Karine Chevreul

**Affiliations:** 1 ECEVE, UMRS 1123, Université Paris Diderot, Sorbonne Paris Cité, INSERM, Paris, France; 2 AP-HP, URC Eco, Paris, France; 3 National Conservatory of Arts and Crafts, LIRSA, 4603 Paris, EA France; 4 World Health Organization Collaborating Centre for Research and Training in Mental Health, Lille, France

**Keywords:** Geographic variations, Psychiatry, Involuntary care, Supply

## Abstract

**Background:**

Involuntary psychiatric care remains controversial. Geographic disparities in its use can challenge the appropriateness of the care provided when they do not result from different health needs of the population. These disparities should be reduced through dedicated health policies. However, their association with the supply of health and social care, which could be targeted by such policies, has been insufficiently studied. Our objectives were therefore to describe geographic variations in involuntary admission rates across France and to identify the characteristics of the supply of care which were associated with these variations.

**Methods:**

Involuntary admission rate per 100,000 adult inhabitants was calculated in French psychiatric sectors’ catchment areas using 2012 data from the national psychiatric discharge database. Its variations were first described numerically and graphically. Several factors potentially associated with these variations were then considered in a negative binomial regression with an offset term accounting for the size of catchment areas. They included characteristics of the supply of care (public and private care, health and social care, hospital and community-based care, specialised and non-specialised care) as well as adjustment factors related to epidemiological characteristics of the population of each sector’s catchment area and its level of urbanization. Such variables were extracted from complementary administrative databases. Supply characteristics associated with geographic variations were identified using a significance level of 0.05.

**Results:**

Significant variations in involuntary admission rates were observed between psychiatric sectors’ catchment areas with a coefficient of variation close to 80%. These variations were associated with some characteristics of the supply of health and social care in the sectors’ catchment areas. Notably, an increase in the availability of community-based private psychiatrists and the capacity of housing institutions for disabled individuals was associated with a decrease in involuntary admission rates while an increase in the availability of general practitioners was associated with an increase in those rates.

**Conclusions:**

There is evidence of considerable variations in involuntary admission rates between psychiatric sectors’ catchment areas. Our results provide lines of thoughts to reduce such variations, in particular by supporting an increase in the availability of upstream and downstream care in the community.

**Electronic supplementary material:**

The online version of this article (10.1186/s12913-018-3064-3) contains supplementary material, which is available to authorized users.

## Background

Involuntary psychiatric care is a form of care where patients’ consent is not required. It is provided to deal with crisis situations where the patient, who is no longer able to make an informed decision, represents a significant danger to oneself or to others due to his/her psychiatric condition [1, 2]. Involuntary care is however controversial as it represents a limitation of one’s freedom and is not retrospectively perceived as justified or beneficial by patients [3]. As a consequence, it is often considered an indicator of the quality of care [4–6] and its reduction is supported by international recommendations [7]. Reforms have therefore been issued to moderate its frequency [1, 8].

Previous research has shown significant geographical variations in involuntary care both between and within countries [1, 5, 9]. Some of these variations arose from different population profiles. The health needs of the population, approximated by the prevalence of severe mental disorders such as psychotic disorders [5], were in particular found to be associated with involuntary care, and so were socio-economic characteristics of the population, including social support and deprivation [10, 11]. In addition, a large body of literature has underscored the possibility that health care supply could also be associated with geographic variations in the use of care [12–14]. A few studies have suggested that this could indeed be the case for variations in the use of involuntary care [5, 10, 15, 16]. However, they have only focused on limited types of providers or on too few areas [5, 9]. Additional work carried out on a large scale and including a wide number of variables is therefore necessary to better illustrate geographical variations in involuntary care and to understand their associations with the supply of care. This is particularly important as disparities in the use of involuntary care – when they do not result from different health needs – can challenge the quality, equity and efficiency of the care provided.

Given these elements, our objective was first to describe geographic variations in involuntary admission rates across France, and second to identify the characteristics of the supply of care which were associated with these variations after adjusting for other relevant factors, in particular population characteristics.

## Methods

### Setting

In France, public mental health care – the only type of care allowed to provide involuntary treatment – is organised separately for adults, children and adolescents, and forensic patients. For these three populations, there is a territorial organization of care which is divided into geo-demographic areas (sectors’ catchment areas) where multidisciplinary teams (sectors) provide integrated outpatient and inpatient care necessary to cover the mental health needs of their population, including preventive, diagnostic and therapeutic services [17]. The staff needed to provide these services is employed by a hospital which can be in charge of several sectors. This hospital may be either public or private non-profit, and it can also be either specialised in psychiatry or a general hospital with an activity in psychiatry which then fulfils the same tasks as a specialised hospital. Additionally, complementary care may be delivered in sectors’ catchment areas by social institutions or private providers, either specialised (such as community-based private psychiatrists, psychologists or private for-profit hospitals) or not (such as general practitioners).

Due to these specificities, our study was carried out in adult non-forensic sectors’ catchment areas, which were our units of analysis, in the entire territory of mainland France. On this territory, involuntary care is regulated by the same national law which states that involuntary care may only be provided by sectors linked to hospitals mandated to do so by regional health agencies [18]. We therefore only analysed data from sectors linked to such hospitals. Furthermore, to ensure comparability and data quality, we only included data from sectors linked to hospitals which provided exhaustive information on their admissions. These sectors were identified by cross-checking aggregated data from the French national discharge database which contains individual information on the use of psychiatric care (*Recueil d’informations médicalisé en psychiatrie*, RIM-P) [19] with data from the annual national survey on health care providers (*Statistique annuelle des établissements de santé*, SAE) [20] (see Additional file 1 for more information on these databases).

As the geographical boundaries of psychiatric sectors’ catchment areas are not publicly available on a national scale and to take into account actual patients’ behaviours when seeking care, we built sectors’ catchment areas using patient-origin data [21]. Based on the official age limit of adult psychiatry in France [22], the zip codes of patients over 16 seen in each sector were extracted from the RIM-P database. Access to individual information from this database is regulated by the French data protection authority (CNIL), which granted us an authorization in August 2014 (Decision DE-2014-090). A geographic information system (Geoconcept® software) was then used to map the catchment areas and to exclude outlier zip codes.

### Involuntary admission rate

The number of inhabitants of each sector’s catchment area admitted in involuntary care at least once in each sector over the course of the year studied (2012) was extracted from the RIM-P database. We only considered involuntary admissions in full-time inpatient care (day and night) provided in hospital settings.

We included involuntary admissions of patients who were diagnosed with a mental disorder from Chapter V of the International Classification of Diseases, tenth revision (ICD-10) [23]. We excluded patients suffering from organic mental disorders, mental retardation and disorders of psychological development (apart from pervasive developmental disorders) because of the specificity of the care they require. This diagnosis scope corresponds to psychiatrists’ expertise in France and has been used in previous international studies in the mental health field [24, 25].

To calculate the involuntary admission rate per 100,000 inhabitants, the number of inhabitants involuntarily admitted at least once over the course of the year 2012 was divided by the total number of inhabitants over 16 in the sector’s catchment area.

### Potential factors associated with geographic variations in involuntary admission rate

#### Factors related to the supply of health and social care

We first considered the supply of public mental health care in sectors’ catchment areas. We included both institutional characteristics (such as participation to teaching activities) and organizational characteristics (such as number of psychiatric inpatient beds or full-time equivalents allocated to psychiatry) of the hospital to which each psychiatric sector was linked.

Second, we considered the supply of private mental health care in sectors’ catchment areas. We included the availability of self-employed community-based psychiatrists or psychologists and of hospitalization beds in private psychiatry.

Third, we considered the availability of non-specialised health care, both for primary care (general practitioners) and for hospital-based care (non-psychiatric hospitalization beds).

Finally, we included data on the supply of social care (residential care or services for disabled individuals).

The full list of supply factors considered is available in Table 1. Information regarding these variables were extracted from administrative databases: the SAE database, the French national database of permanent facilities (*Base permanente des équipements*), the national register of health and social institutions (*Fichier national des établissements sanitaires et sociaux*) and the national directory of professionals [20, 26–28] (see Additional file 1 for more information on these databases).

**Table 1:** Variations in involuntary admission rates between psychiatric sectors’ catchment areas

	Mean (SD)	Median (interquartile range)	Range	CV (%)	Ratio 90/10th percentiles
Involuntary admission rate per 100,000 inhabitants	21.92 (17.43)	17.51 (21.98)	135.11	79.51	10.24

#### Epidemiological factors

We considered several direct characteristics of population health needs. They included three characteristics of the mental health status of the population living in each sector’s catchment area. They were the rate of individuals suffering from chronic psychiatric disorders, assessed by the number of individuals covered by the long-term illness scheme for psychiatric reasons (i.e. individuals who are exonerated from co-payments of any health care linked with their chronic illness) per 100,000 inhabitants; the percentage of deaths by suicide; and the acute admission rate for psychiatric disorders (including both voluntary and involuntary admissions). We also included characteristics of the overall health status of the population (global mortality rate, rate of individuals suffering from chronic somatic disorders, and acute admission rate for somatic disorders). These variables were extracted from the database of the national centre on epidemiological causes of death (*Centre d’épidémiologie sur les causes médicales de décès*), the census database (*Base des recensements de la population*), the French national discharge database for somatic care (*Programme de médicalisation des systèmes d’information en médecine, chirurgie, obstétrique*) and the Eco-Santé database which provides data on the health of the French population before 2016 [29–32] (see Additional file 1 for more information on these databases).

Additionally, we considered the demographics of the population, which have been shown to be correlated with health needs [33]: the mean age of the adult population in the sectors’ catchment areas and the percentage of women in these areas extracted from the census database (Additional file 1) [31].

Finally, socio-economic factors have also been shown to be correlated with health needs [34, 35]. We therefore calculated a proxy deprivation index for each zip code belonging to a sector’s catchment area and calculated its mean value in the area, using a validated composite index specifically developed for France, the FDep. This index takes into account the median household income, the percentage of high school graduates in the population aged 15 years and older, the percentage of blue-collar workers in the active population and the unemployment rate [36–38].

#### Level of urbanization

Urbanicity is likely to be linked with involuntary care through different mechanisms related to coordination and distances between the different types of care supply [39] and the availability of other actors which can play a role in involuntary care, such as police stations. We therefore introduced the level of urbanization as an additional adjustment factor. This characteristic was assessed by the density of inhabitants in the zip codes of the sectors’ catchment areas and extracted from a national administrative database on urbanicity (*Base des unités urbaines*) [40] (see Additional file 1).

### Methods of analysis

#### Description of variations

We first described the characteristics of the population in sectors’ catchment areas either by the mean and standard deviation (SD) or by number and percentage.

Variations in involuntary admission rates between catchment areas were described by calculating the national mean, SD, median, interquartile range and range. A coefficient of variation (CV), which measures the dispersion around the national mean [41], was also calculated. This coefficient was interpreted together with the ratio between the 90th and the 10th percentiles of the distribution, which is less sensitive to outlier values (a high value for this ratio suggests that variations remain when extreme observations are excluded from the analysis) [13]. To explore further the potential impact of outlier values, we constructed a waterfall plot representing the involuntary admission rate in each sector’s catchment area, ranked by decreasing order, in comparison to the national average.

#### Identification of factors associated with variations in involuntary admission rates

To identify the characteristics of the supply of health and social care in sectors’ catchment areas which were associated with variations in involuntary admission rates, we carried out a negative binomial regression to account for the overdispersion of data (deviances considerably exceeding the degrees of freedom). The dependent variable was the observed number of inhabitants of each sector’s catchment area admitted in involuntary care at least once over the course of the year 2012 (event) while the natural logarithm of the total number of inhabitants aged over 16 in each sector’s catchment area was used as an offset term in the regression [42–44]. All supply factors (described above) were introduced as explanatory variables (except for explanatory variables that were highly correlated). We also added relevant adjustment factors relating to epidemiological data and level of urbanization. Statistical significance was taken to be indicated by a probability value of 0.05 or less. The analysis produced estimated values of the regression coefficients for all explanatory variables in the model, whose sign indicated whether the association was positive or negative, as well as their 95% confidence intervals (95%CI). Regression coefficients for each explanatory variable were finally exponentiated to obtain an estimation of the inpatient admission incidence rate ratio given the other variables were held constant in the model. The rate ratio for the dependent variable was expressed for a one unit increase in each continuous explanatory variable while, for categorical explanatory variables, a reference was chosen for comparison [42, 43].

All the analyses were performed using SAS software version 9.4 (SAS Institute Inc., Cary, NC, USA).

## Results

### Setting

Five hundred fourteen adult psychiatric sectors were linked to hospitals providing exhaustive information on their admissions. They accounted for 66.0% of all adult non-forensic psychiatric sectors delivering involuntary care reported in the RIM-P database for the year 2012 in mainland France. They corresponded to 168 hospitals representing 73.4% of all hospitals participating in psychiatric sectorisation and mandated by regional health agencies to deliver involuntary care. Sectors that had data included were linked to hospitals which were more often public than hospitals linked to excluded sectors (96.6% vs. 90.3%, *p* = 0.043), but they did not differ in terms of other main institutional, organizational or case-mix characteristics.

### Characteristics of the population of sectors’ catchment areas

The mean percentage of women of sectors’ catchment areas was 52.3% (±2.5) and the mean age of this population was 48.1 years old (±3.6). On average, the number of individuals suffering from chronic mental disorders per 100,000 inhabitants of sectors’ catchment areas was 1627.4 (±366.1) and deaths by suicide represented 5.1% (± 2.0%) of all deaths.

Five hundred fifty-seven thousand five hundred forty-three individuals living in these catchment areas were treated in the corresponding psychiatric sectors in 2012 (accounting for 5,514,001 admissions). Forty thousand four hundred seventeen individuals were admitted at least once in involuntary care. They represented 5.7% of all patients seen in included psychiatric sectors and 29.1% of patients admitted to inpatient care in these sectors.

### Variations in involuntary admission rates

The national mean psychiatric involuntary admission rate was 21.9 (±17.4) per 100,000 inhabitants of a sector’s catchment area. Significant variations were observed between catchment areas with a coefficient of variation close to 80%. These variations were not only a result of catchment areas with outlier values, as the ratio between the 90th and the 10th percentiles of the distribution was superior to ten (Table 1).

**Fig. 1:**
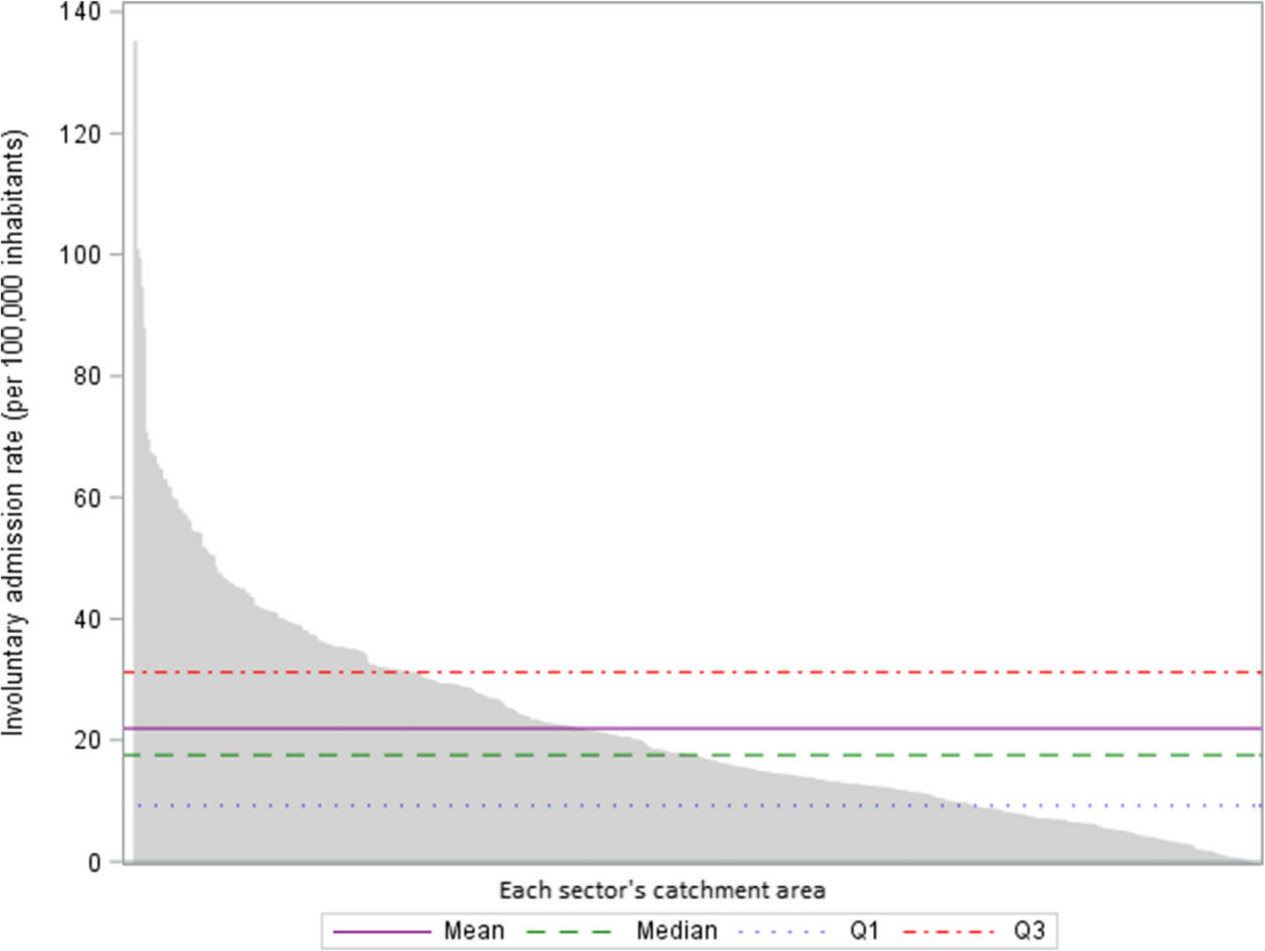
Involuntary admission rate in the catchment area of each sector in comparison to the national average. Q1: upper limit of the first quartile; Q3: lower limit of the last quartile

Similarly, the waterfall plot showed a wide scattering of the values of the involuntary admission rate in the different catchment areas (not only for a limited number of catchment areas) in comparison to aggregated national values (Fig. 1).

### Factors associated with variations in involuntary admission rates

Thirteen characteristics of the supply of health and social care in sectors’ catchment areas were introduced in the model after controlling for potential correlations (see Table 2 and associated footnotes). After adjusting for epidemiological data and level of urbanization, several of these characteristics were significantly associated with the involuntary admission rate.

**Table 2:** Results of the negative binomial regression

Variable	Estimated value of the coefficient	Standard error	95% CI of the coefficient		Exponentiated coefficient	*P*-value
			Lower bound	Upper bound		
Intercept	−6.2746	1.1268	−8.4831	−4.0662		< 0.0001
Characteristics of the supply of health and social care						
Supply of public mental health care						
Characteristics of the hospital to which each sector was linked						
Private non-profit (vs. public)	−0.1543	0.2249	− 0.5951	0.2864	0.8570	0.4926
Participation to teaching activities (vs. no participation)	−0.2567	0.1128	−0.4778	−0.0355	0.7736	0.0229
Specialization in psychiatry (vs. general hospital)	0.1960	0.0841	0.0312	0.3607	1.2165	0.0197
Participation to emergency care (vs. no participation)	0.1500	0.1241	−0.0933	0.3933	1.1618	0.2269
Number of inpatient beds per 100,000 inhabitants^c^	− 0.0027	0.0029	− 0.0085	0.0030	0.9973	0.3525
Supply of private mental health care (per 100,000 inhabitants)						
Number of community-based private psychiatrists^b^	−0.0113	0.0065	−0.0239	0.0014	0.9888	0.0310
Number of psychologists	0.0011	0.0010	−0.0008	0.0030	1.0011	0.2644
Number of hospitalization beds of private psychiatry^a^	0.0011	0.0024	−0.0036	0.0058	1.0011	0.6544
Supply of non-specialised care (per 100,000 inhabitants)						
Number of general practitioners^b^	0.0047	0.0027	−0.0006	0.0099	1.0047	0.0499
Number of non-psychiatric hospitalization beds	−0.0001	0.0001	−0.0003	0.0001	0.9999	0.3535
Supply of social care (per 100,000 inhabitants)						
Number of beds in housing institutions for disabled individuals	−0.0017	0.0007	−0.0020	0.0007	0.9983	0.0466
Capacity of centres providing care through employment	−0.0015	0.0008	−0.0030	0.0000	0.9985	0.0548
Capacity of housing and social rehabilitation centres	−0.0008	0.0010	−0.0026	0.0011	0.9992	0.4305
Epidemiological characteristics						
Psychiatric health status of the population						
Number of individuals suffering from chronic mental disorders (per 100,000 inhabitants)^a^	0.0000	0.0002	−0.0003	0.0003	1.0000	0.9719
Percentage of deaths by suicide among total deaths	−0.0193	0.0333	−0.0846	0.0460	0.9809	0.5623
Acute admission rate for psychiatric disorders (per 100,000 inhabitants)	0.0127	0.0009	0.0109	0.0144	1.0128	< 0.0001
Overall health status of the population (per 100,000 inhabitants)						
Acute admission rate for somatic disorders	0.0000	0.0000	−0.0001	−0.0000	1.0000	0.0041
Mortality rate	−0.0007	0.0010	−0.0027	0.0013	0.9993	0.4665
Number of individuals suffering from chronic somatic disorders	0.0000	0.0000	−0.0001	0.0001	1.0000	0.9070
Demographics of the population						
Number of women (per 100,000 inhabitants)	0.0000	0.0000	−0.0000	0.0001	1.0000	0.0566
Mean age of individuals aged over 16	−0.0650	0.0187	−0.1017	−0.0284	0.9371	0.0005
Socio-economic characteristics of the population						
Quintile of the mean deprivation index (FDep) (from lower to higher deprivation), reference: 5th quintile						
1	−0.2945	0.1606	−0.6092	0.0202	0.7449	0.0666
2	−0.0920	0.1267	−0.3403	0.1564	0.9121	0.4679
3	−0.0592	0.1242	−0.3027	0.1842	0.9425	0.6333
4	−0.0864	0.1206	−0.3229	0.1500	0.9172	0.4737
Level of urbanisation						
Quantile of the level of urbanisation (from lower to higher urbanisation), reference: 6th quantile						
1	0.0701	0.1253	−0.1754	0.3156	1.0726	0.5758
2	0.0423	0.1613	−0.2739	0.3585	1.0432	0.7933
3	0.1125	0.3969	−0.6655	0.8904	1.1191	0.7769
4	0.5827	0.5735	−0.5412	1.7067	1.7909	0.3095
5	0.0099	0.1254	−0.2359	0.2557	1.0099	0.9373

^a^and ^b^Significant correlations were observed between these variables. However, corresponding correlation coefficients were weak and there were strong hypotheses on associations of these variables with involuntary admission rates so they were all introduced in the model

^c^The number of inpatient beds per 100,000 inhabitants of the catchment area was highly correlated with the total number of full-time equivalents per 100,000 inhabitants allocated to psychiatric care by the hospital to which each sector was linked (ρ = 0.96; *p* < 0.0001). We therefore only introduced the number of beds in the model

Regarding the characteristics of public mental health care, the fact that a sector was linked to a hospital specialised in psychiatry (vs. a general hospital) was associated with a 21.7% increase in the involuntary admission rate per 100,000 inhabitants of the catchment area. The fact that a sector was linked to a hospital participating to teaching activities (vs. a hospital not participating to such activities) was associated with a 22.6% decrease in this rate (Table 2).

Considering the characteristics of private health care, an increase by 1.0 in the number of community-based private psychiatrists per 100,000 inhabitants, whose average national value was 12.8, was associated with a decrease by 1.1% of the involuntary admission rate. On the contrary, an increase by 1.0 in the number of general practitioners (GPs) per 100,000 inhabitants (average national value = 108.2) was associated with an increase by 0.5% of the involuntary admission rate (Table 2).

Regarding the availability of social care, an increase by 1.0 in the number of beds in housing institutions for disabled individuals per 100,000 inhabitants (average national value = 195.4) was associated with a decrease by 0.2% of the involuntary admission rate (Table 2).

Notably, some of our adjustment factors, in particular the socio-economic characteristics of the population and the level of urbanization, were not significantly associated with involuntary admission rates in the multivariate analysis (Table 2).

## Discussion

Significant variations in involuntary admission rates were observed between sectors’ catchment areas in France. After adjusting for epidemiological differences and varying levels of urbanization, an increase in the supply of health and social care, in particular the availability of community-based private psychiatrists and the capacity of housing institutions for disabled individuals, was associated with a decrease in involuntary admission rates. In contrast, an increase in the availability of general practitioners was associated with an increase in involuntary admission rates. Finally, these rates also increased in the catchment areas of sectors linked to a hospital specialised in psychiatry (vs. a general hospital) while they decrease in the catchment areas of sectors linked to a hospital participating to teaching activities.

While deaths by suicide in France account for a particularly high share of the total mortality [45], we believe that our population of interest is relatively comparable to that of other countries with similar economic developments in terms of prevalence of mental disorders and demographic structure [45–48]. The national mean involuntary admission rate (22 per 100,000 inhabitants) found in our study was close to that observed in other European countries, such as Denmark or Italy [49, 50], even though the percentage of involuntary admissions over the total number of inpatient admissions seemed to be higher than in other countries [5]. In addition, these national estimates were older than ours and comparisons must be made with caution. A more recent study carried out at the local level in Italy showed wide variations in involuntary admission rates between geo-demographic areas, similarly to what was observed between French psychiatric sectors’ catchment areas. However, the variations seemed to be slightly less considerable than in our study (CV of 69% vs. 80%) [5]. These differences may be partly explained by a stronger and older consensus on the benefits of deinstitutionalization in Italy [51], a more homogenous development of alternatives in the community as a consequence, and the scale of the study (regional vs. national).

The extent of the variations observed between psychiatric sectors’ catchment areas questions the adequacy of care and suggests that some of them may be unwarranted. This hypothesis is further supported by the associations found between these variations and the characteristics of the supply of health and social care in sectors’ catchment areas, even after adjusting for epidemiological differences and varying levels of urbanization. If previous research has shown that the availability of social workers and of less restrictive forms of care were associated with involuntary care [5, 52, 53], no study has ever considered as wide a range of supply factors, which limits comparisons.

Regarding the underlying mechanisms which could explain the associations found in our study, several hypotheses can be made. First of all, it could be hypothesized that supply influences demand. This may explain that involuntary admission rates are higher in the catchment areas of sectors linked to psychiatric hospitals compared to those linked to general hospitals, as the former are more likely to have high-capacity closed wards. It may additionally result from varying practice patterns between the different types of hospital, which could also explain that involuntary admission rates are lower in the catchment areas of sectors linked to a hospital participating to teaching activities. These varying practice patterns should be further explored, particularly by qualitative studies. Second, it could be hypothesized that the availability of both upstream and downstream care in the community (especially community-based private psychiatrists and residential alternatives) enables a better continuity of care which reduces crisis situations and the need for involuntary care. This hypothesis does not hold for general practitioners whose availability was associated with an increase in involuntary care. However, the difficulties of French GPs to adequately detect the mental health needs of their patients have often been underscored while they also lack appropriate training to address such needs once identified [54, 55]. In addition, it could be linked to non-optimal collaborations between primary care and psychiatric sectors [56]. France is indeed one of the European countries where GPs address the least often their patients to specialised psychiatric care [57]. In parallel, GPs also report difficult relationships with sectors as they estimate that they do not receive enough information on their patients’ evolution when they are seen in public psychiatry [58]. Finally, it is possible that part of the association found between the availability of GPs and the involuntary admission rate translate unmeasured differences in the level of urbanisation between sectors’ catchment areas. Our indicator of urbanisation was indeed only based on population density, which could explain that it was not associated with involuntary care, similarly to what was observed in a previous study using the same kind of indicator of urbanisation [5]. Complementary quantitative and qualitative studies would be particularly useful in exploring further these hypotheses.

This study, conducted in France, is the first to focus on geographical variations in involuntary care in a whole country. We considered a wide range of supply factors, relating to both public and private care, health and social care, hospital and community-based care, and specialised and non-specialised care, by mobilizing ten complementary national administrative databases, mostly compiled by governmental institutions. Studies available in the current literature focused on a limited geographic area and included few characteristics of the supply of care available in the environment [5, 9]. While carrying out analysis at the local level can increase the availability and accuracy of data, it often impairs the generalizability of the findings, especially as the supply of care is more likely to be homogenous within a given region than between regions. The results of our study should nevertheless be interpreted in light of several caveats. First of all, it should be kept in mind that we did not aim to explain use of involuntary care by individual patients. We adopted an ecological and geographical approach and used sectors’ catchment areas as our units of analysis. It should therefore not be assumed that individual mental health care users have characteristics similar to the population average in sectors’ catchment area. Concurrently, the significant associations found in our study could be affected by compositional effects which we were not able to account for in our analysis. Further research could be useful to explore such effects by calculating inpatient involuntary admissions rates directly standardised on population demographic characteristics in each sector’s catchment area prior to conducting the multivariable analysis. Second, the RIM-P database, from which we extracted data on inpatient involuntary admissions, is not used for financial or certification purposes and this can limit data quality. In order not to interpret the variability in data reporting and quality as variability in inpatient involuntary admission rates, we therefore had to exclude the data of around one third of the sectors meeting our inclusion criteria as they were linked to hospitals for which exhaustive information on their admissions were not available. However, such hospitals presented very few differences with hospitals whose data was included. Third, we were limited by the availability of some of the potential factors associated with variations in psychiatric care due to our use of administrative databases. Regarding supply factors, previous research focusing on the local context of mental health care collected exhaustive data on available primary, secondary and tertiary health care supply, as well as on all social and voluntary services through ad hoc interviews with local stakeholders and a validated taxonomy which facilitates international comparisons [59, 60]. These kinds of approaches could be usefully implemented for the study of geographic variations in mental health care use. Nevertheless, their feasibility is limited on the national scale. Furthermore, additional epidemiological data, used here as adjustment factors, could be considered. Indeed our study is notably impaired by the lack of detailed information related to the prevalence of the different mental disorders in sectors’ catchment areas; such information is currently not available. Similarly, no information is collected on ethnicity and immigration status of the population in France [61], while these characteristics could also be correlated with mental health needs.

## Conclusions

Our results underscore the need to routinely monitor geographic variations in psychiatric involuntary care as they remain considerable. Our findings also suggest several measures that can be explored by policy makers to target unwarranted variations in involuntary care worldwide, notably the development of upstream and downstream care in the community. In France, an increase in the availability of community-based private psychiatrists through their better repartition is a lever which could be mobilized. This has already been advocated by international recommendations which support a homogenous geographical distribution of mental health professionals, in line with population needs, and equal access to mental health care for all [62, 63]. An increase in the availability of downstream alternatives to hospitalization such as housing institutions for disabled individuals could also be planned.

## Additional file

Additional file 1: Description of the different databases used in the study. Table including the name, main content and data compilation method for each database included in the study. (DOCX 17 kb)

## Abbreviations

95% CI: 95% confidence intervals
CNIL: French data protection authority
CV: Coefficient of variation
FDep: French deprivation index
GP: General practitioner
ICD-10: International classification of diseases, tenth revision
Q1: Upper limit of the first quartile
Q3: Lower limit of the last quartile
RIM-P: Recueil d’informations médicalisé en psychiatrie, French national psychiatric discharge database
SAE: Statistique annuelle des établissements de santé, French annual national survey on health care providers
SD: Standard deviation
