# Supplementary material for: Geographic variations in involuntary care and associations with the supply of health and social care: results from a nationwide study
Source: BMC Health Serv Res. 2018 Apr 6;18:253. doi: 10.1186/s12913-018-3064-3 (PMC5889610; doi:10.1186/s12913-018-3064-3)
Supplement: Supplementary file 1 — Description of the different databases used in the study. Table including the name, main content and data compilation method for each database included in the study. (DOCX 17 kb) [file 12913_2018_3064_MOESM1_ESM.docx]

**Additional file 1. Description of the different databases used in the study**

| **Name** | **Main content** | **Data compilation** |
| --- | --- | --- |
| French national discharge database for psychiatric care (*Recueil d’informations médicalisé en psychiatrie*, RIM-P) | Service utilization data for inpatient and outpatient psychiatric care delivered by hospitals, limited clinical and demographics information on patients. | Data collected through hospitals’ information system with some *a posteriori* controls from the institution in charge of compiling the data, the Technical agency for information on hospital care (*Agence technique de l’information sur l’hospitalisation*, ATIH). Data not used for financial or certification purpose. |
| French national survey on health care providers (*Statistique annuelle des établissements de santé*, SAE) | Organisational characteristics of French hospitals and aggregated service utilization data in these hospitals. | Exhaustive and compulsory annual survey among all hospitals compiled by the French ministry of health. |
| French national register of health and social institutions (*Fichier national des établissements sanitaires et sociaux*, FINESS) | Identification and institutional characteristics of French hospitals. | Data updated annually by the French ministry of health. |
| French national database of permanent facilities (*Base permanente des équipements*) | Indicators of facilities and services (including health and social care supply) available for the population of a communal or infracommunal territory. | National institute for statistical and economic studies (*Institut national de la statistique et des études économiques*, INSEE). |
| French national directory of professionals (*Annuaire pages jaunes*) | List and address of professionals by type of professionals. | Data compiled by a private company (SoLocal group). |
| Database of the French national centre on epidemiological causes of death (*Centre d’épidémiologie sur les causes médicales de décès*) | Causes of death of individuals coded according to the ICD-10 aggregated by places of residence. | Data from death certificates compiled and cross-checked by the Centre on epidemiological causes of death (*Centre d’épidémiologie sur les causes médicales de décès*, CépiDc) which is part of the French national institute for health and medical research. |
| French national discharge database for somatic care (*Programme de médicalisation des systèmes d’information en médecine, chirurgie, obstétrique*, PMSI-MCO) | Service utilization data for inpatient and outpatient care for general somatic care, surgery and obstetrics delivered by hospitals, limited clinical and demographics information on patients. | Data collected through hospitals’ information system with some *a posteriori* controls from the institution in charge of compiling the data, the Technical agency for information on hospital care (*Agence technique de l’information sur l’hospitalisation*, ATIH). Data used for financial purpose in the frame of an activity-based payment model. |
| Eco-Santé database | Data on the health status of the population, health spendings, demography, social welfare, etc. | Data originating from different institutional sources and compiled by the Institute for research and information in health economics (*Institut de recherche et documentation en économie de la santé*, IRDES) until April 2016. |
| French national census database (*Base des recensements de la population*) | Data on demographics and socio-economic characteristics of the population of a given geographic area. | Data is collected and compiled by the National institute for statistical and economic studies (*Institut national de la statistique et des études économiques*, INSEE) |
| French national database on urbanicity (*Base des unités urbaines*) | Data on the level of urbanization of the different geographic areas of the French territory. | Data is compiled by the National institute for statistical and economic studies (*Institut national de la statistique et des études économiques*, INSEE) |
